# Novel robotic gripper for traction and closure in colorectal endoscopic submucosal dissection

**DOI:** 10.1016/j.vgie.2024.12.004

**Published:** 2024-12-26

**Authors:** Sang Hyun Kim, Hyuk Soon Choi, Han Jo Jeon, Eun Sun Kim, Bora Keum, Yoon Tae Jeen, Sangjeong Ahn, Joo Ha Hwang, Hoon Jai Chun

**Affiliations:** 1 Division of Gastroenterology and Hepatology, Korea University College of Medicine, Seoul, South Korea; 2 Department of Pathology, Korea University Anam Hospital, College of Medicine, Korea University, Seoul, South Korea; 3 Division of Gastroenterology and Hepatology, Stanford University, Stanford, California, USA

Endoscopic submucosal dissection (ESD) is widely recognized as a minimally invasive approach for treating colorectal tumors.^1^ Tissue traction and defect closure are critical components for enhancing the safety and effectiveness of colorectal ESD procedures (Fig. 1).^2^^,^^3^ A novel robotic gripper has recently been developed that effectively combines traction and closure functions (ROBOPERA-TraCloser [Dual Gripper]; ENDOROBOTICS, Seoul, South Korea) (Fig. 2). We present the application of this device and demonstrate its utility in colorectal ESD through 2 case studies.

**Figure 1:**
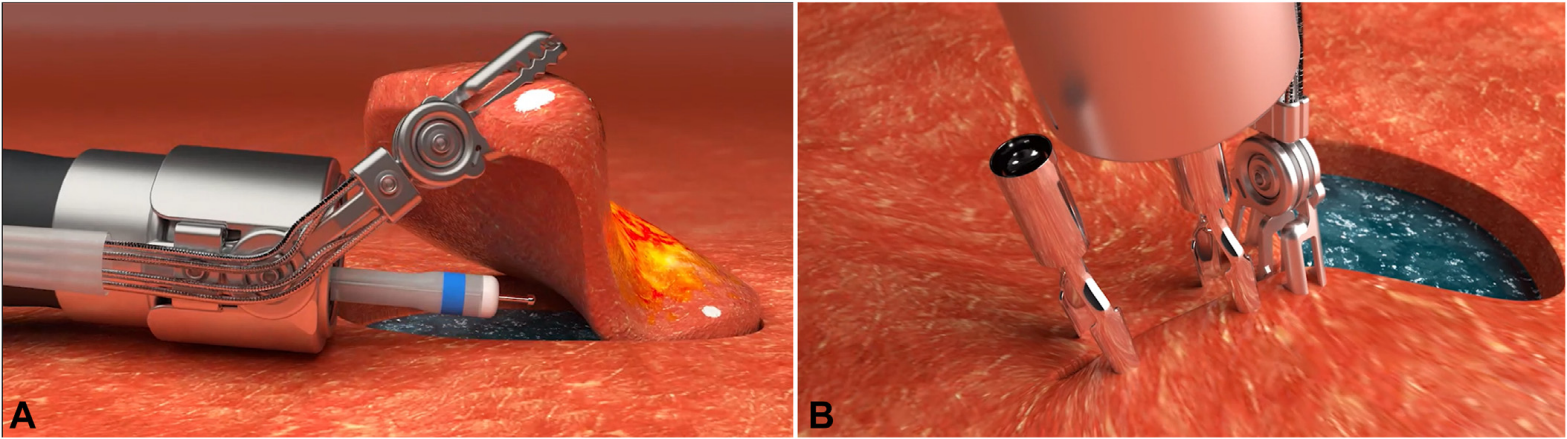
**A,** Schema of ESD using a robotic gripper. One side jaw is opened to grasp the tissue and lift it, thereby exposing the submucosal dissection plane. **B,** Schema of ESD defect closure using a robotic gripper. One jaw of the gripper grasps the ulcer’s edge and pulls it toward the opposite side to facilitate approximation. The other jaw then grasps the opposite edge. Once approximated, the wound is easily closed using conventional through-the-scope clips. *ESD*, Endoscopic submucosal dissection.

**Figure 2:**
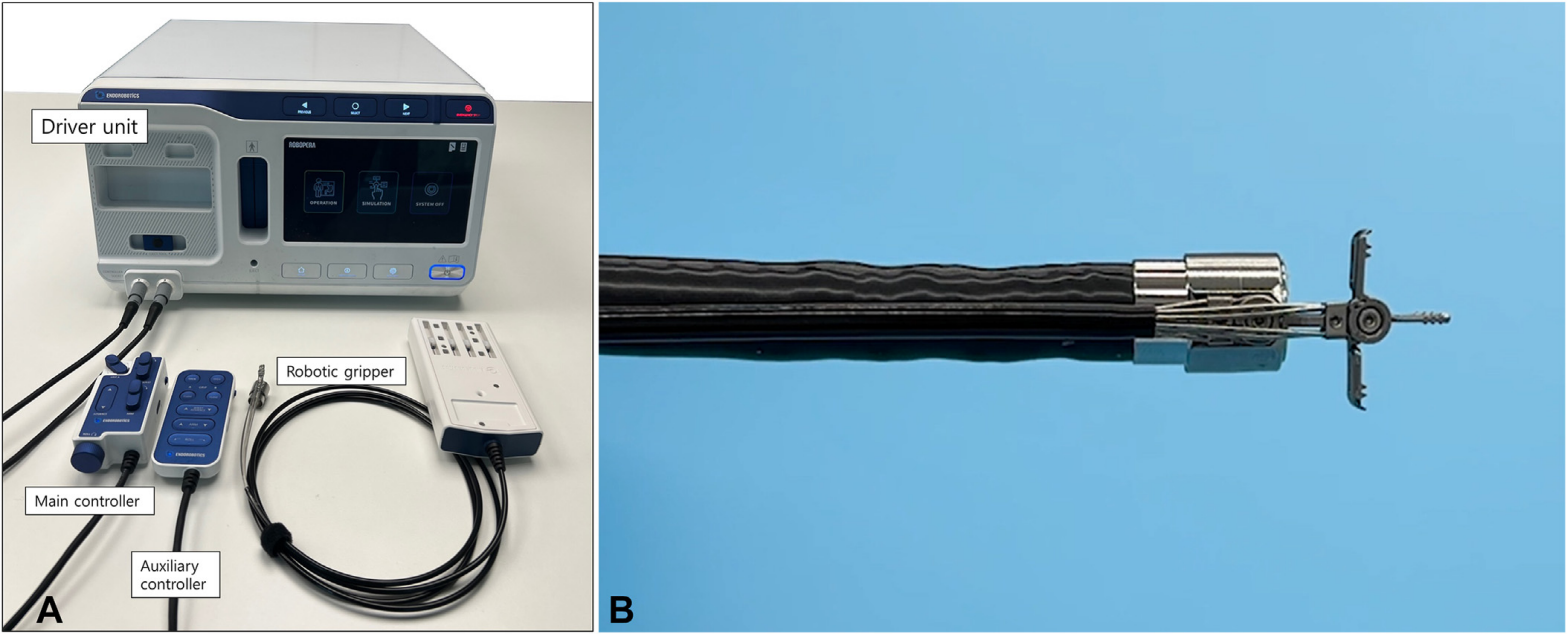
Overview of the robotic traction and closure device. **A,** The device consists of 3 parts: a dual-function robotic gripper, controllers, and a driver unit. The endoscopist uses the main controller to operate the robotic gripper, allowing precise movements in upward, downward, left, right, and 270° circumferential directions along the distal end of the endoscope. The assistant can also use an auxiliary controller to adjust the gripper’s movement. The driver unit acts as the actuator, managing the fine movements of the robotic gripper. To complete the setup, the cartridge connected to the robotic gripper is inserted into the driver unit, and the robotic gripper is attached to the tip of the endoscope. **B,** The robotic gripper features 2 independently operating side jaws and a central fixed support.

## Case

**Figure 3:**
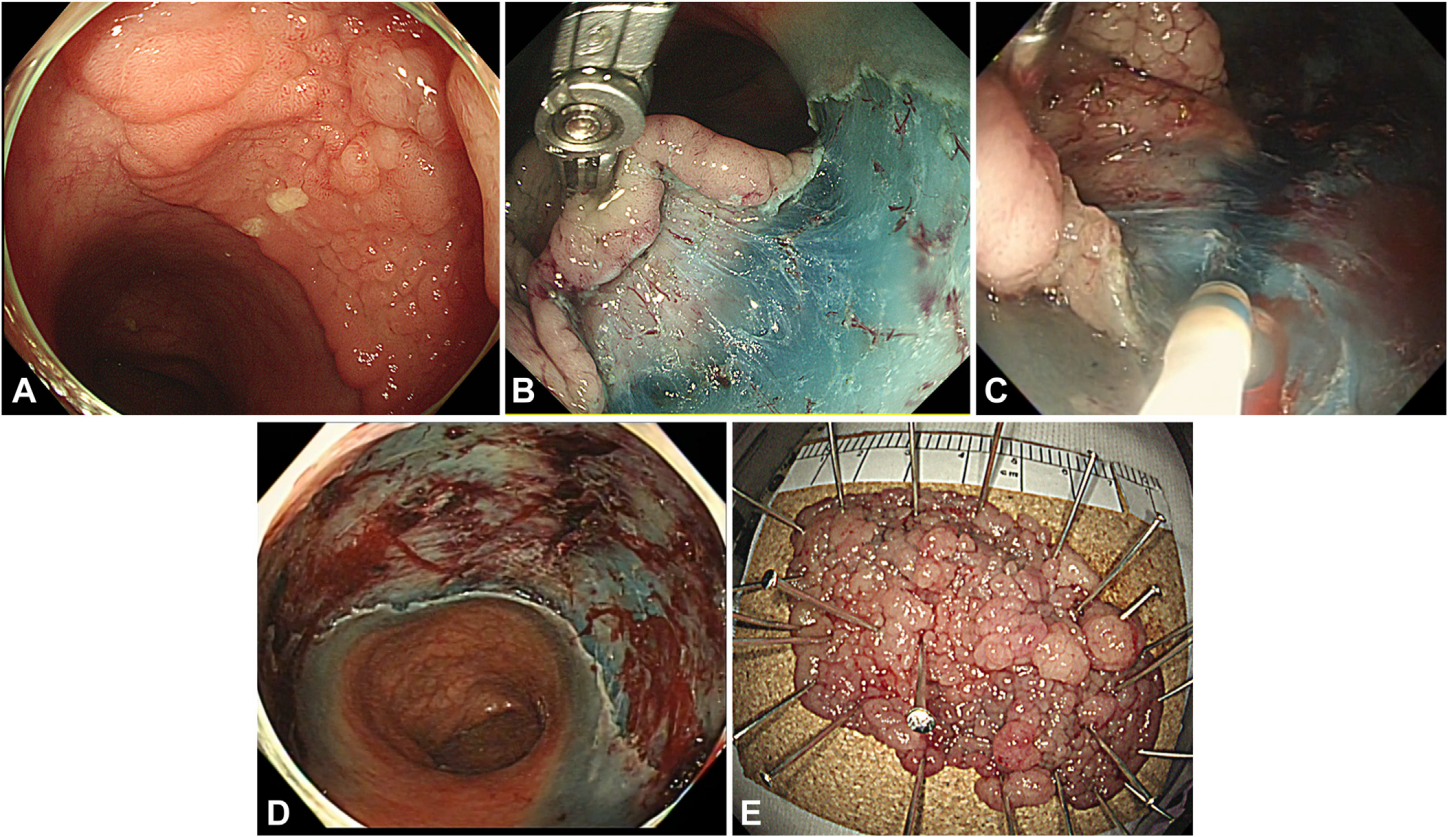
Summary of the first case. **A,** A large LST in the sigmoid colon. Detailed evaluation with high-definition white-light and narrow-band imaging identified the lesion as a granular LST of mixed nodular type, predominantly JNET 2A with the focal JNET 2B pattern. **B,** The proximal edge of the mucosal flap was grasped by the robotic gripper, and the arm was lifted upward to apply the traction to the dissection plane. **C,** The gripper regrasped the flap to further improve visualization of the submucosal plane. As the dissection plane was extended, the traction force was adjusted by reorienting the robotic gripper to enhance exposure of the submucosal tissue. **D,** ESD defect after completion of dissection. **E,** Final resected specimen, measuring 7.1 × 4.0 cm. *ESD*, Endoscopic submucosal dissection; *LST*, laterally spreading tumor.

A 50-year-old woman underwent a colonoscopy, which revealed a large laterally spreading tumor in the sigmoid colon, 19 cm from the anal verge (Fig. 3). On inspection, the lesion was identified as a granular laterally spreading tumor of mixed nodular type, predominantly classified as JNET 2A with the focal JNET 2B pattern. The decision was made to proceed with ESD. The procedure was performed using a conventional gastroscope (GIF-HQ290; Olympus, Tokyo, Japan), an electrocautery knife (DualKnife J; Olympus), and the robotic gripper (TraCloser; ENDOROBOTICS). After creating a flap from the anal side, the robotic gripper, which had been mounted on the tip of the endoscope, was introduced. The proximal edge of the mucosal flap was grasped by the gripper, and the arm was lifted upward to apply the traction to the dissection plane (Fig. 3). During dissection, the robotic arm was adjusted to enhance exposure of the submucosal dissection plane. The procedure achieved complete en bloc resection in 68 minutes. The final specimen measured 7.1 × 4.0 cm, and histologic examination revealed tubulovillous adenoma with high-grade dysplasia (Fig. 3).

**Figure 4:**
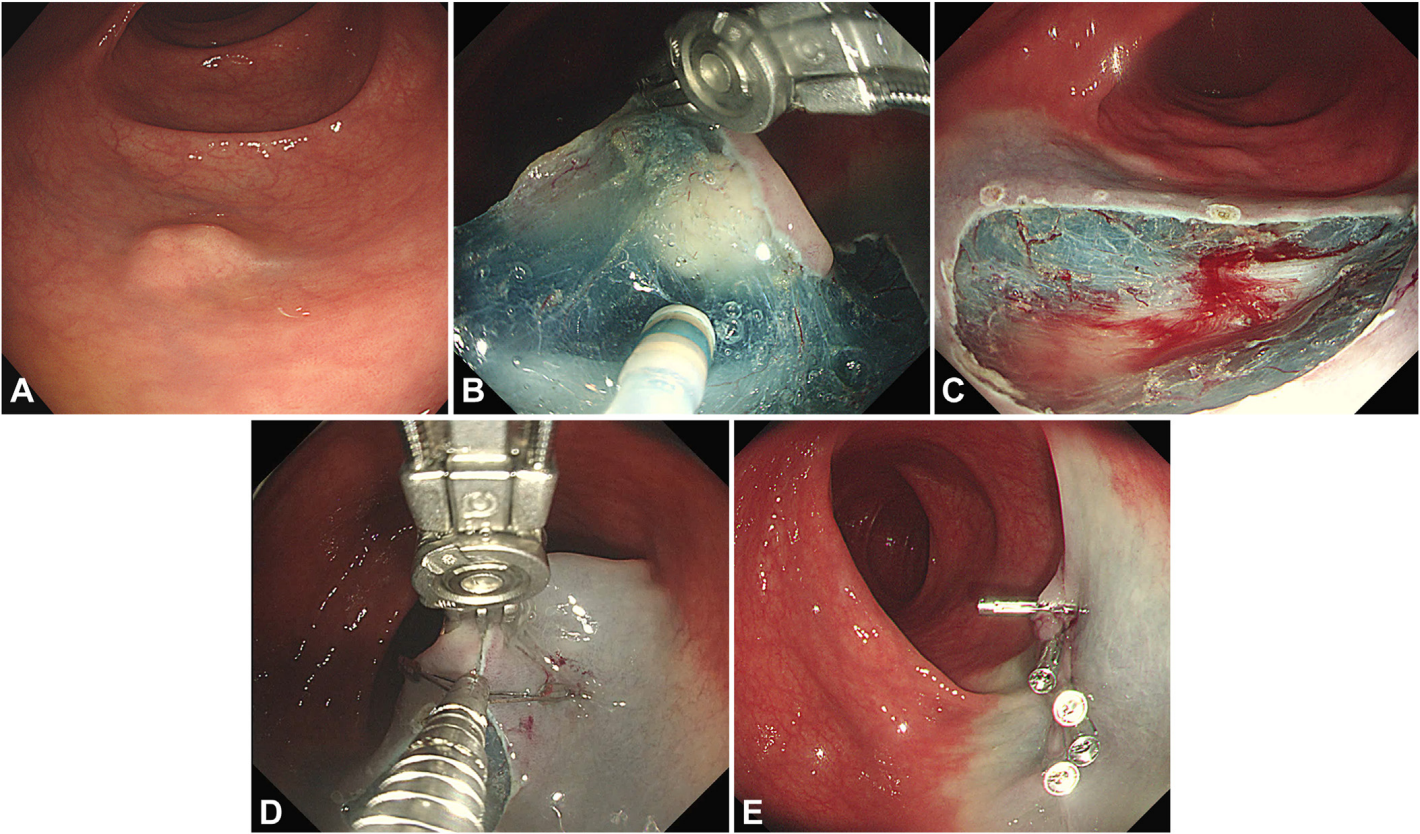
Summary of the second case. **A,** A 1-cm, yellow-colored subepithelial tumor in the mid rectum. **B,** The robotic gripper was used to lift the flap, allowing full visualization of the yellow nodule within the submucosal space. **C,** ESD defect after completion of dissection. **D,** The robotic gripper was used to approximate ESD defect. **E,** Complete closure of ESD defect using TTS clips. *ESD*, Endoscopic submucosal dissection; *TTS*, through-the-scope.

The second case is a 51-year-old man who was referred after a screening colonoscopy revealed a yellow-colored subepithelial tumor in the mid rectum (Fig. 4). A plan for ESD was made under the suspicion of a neuroendocrine tumor. First, a circumferential mucosal incision was performed using a DualKnife. Once the robotic gripper was used to lift the flap, the yellow nodule within the submucosal space became fully visible (Fig. 4). With a clear view of the nodule, the submucosal dissection was safely completed. Complete en bloc resection was achieved in 16 minutes. Because the patient was on anticoagulants, clip-based defect closure was attempted to prevent delayed bleeding. Using the robotic gripper for tissue approximation, we successfully closed the defect with through-the-scope (TTS) clips (Micro-Tech Co, Nanjing, China) in 4 minutes and 40 seconds (Fig. 4). The final specimen measured approximately 2.3 × 1.7 cm, and histologic examination revealed grade 1 neuroendocrine tumor.

## Discussion

As demonstrated in these cases, the primary advantage of this device is its ability to support 2 critical functions of an ESD assistive tool: providing traction and facilitating defect closure. Unlike current traction methods, this device’s ability to instantly grasp and regrasp enables easy application of traction at various points of the lesion throughout the procedure. The robotic gripper is capable of 270° circumferential movement at the tip of the endoscope, allowing for precise adjustment of the traction force throughout the procedure (Video 1, available online at www.videogie.org). After completing the ESD, the gripper can be intuitively used for tissue approximation, allowing for easy closure with TTS clips. This seamless transition from dissection to closure enhances procedural efficiency.

However, the device presents certain limitations. First, after grasping the tissue, the dissection is performed from a fixed position under a distant view, with limited scope maneuverability—a common drawback of similar traction devices.^4^ As the dissection progresses, the mobility of the flap increases, gradually improving maneuverability. As an add-on device, its usability may be limited in narrow working spaces due to its size, and an overtube is necessary for effective access to the right-sided colon.

In conclusion, the robotic gripper provides a promising alternative to conventional traction and closure devices for colorectal ESD, offering potential improvements in safety and procedural efficiency.

## FUNDING INFORMATION

This work was supported by the Technology development Program (RS-2023-00321839) funded by the Ministry of SMEs and Startups (MSS, South Korea) and the National Research Foundation of Korea (NRF) grant funded by the Korean government (MSIT) (RS-2024-00341814).

## Disclosure

Drs Choi, Keum, and Chun hold stocks for ENDOROBOTICS. Dr Hwang is a consultant for Olympus, Medtronic, Boston Scientific, Micro-Tech, Fujifilm, ERBE, LumenDi, Neptune, Noah, and ENDOROBOTICS. The other authors have no conflicts of interest to disclose.

## Supplementary data

Video 1 Demonstration of a novel robotic gripper for traction and closure during colorectal endoscopic submucosal dissection.
